# Relationship between Resilience and Self-regulation: A Study of Spanish Youth at Risk of Social Exclusion

**DOI:** 10.3389/fpsyg.2017.00612

**Published:** 2017-04-20

**Authors:** Raquel Artuch-Garde, Maria del Carmen González-Torres, Jesús de la Fuente, M. Mariano Vera, María Fernández-Cabezas, Mireia López-García

**Affiliations:** ^1^Department of Education and Psychology, International University of La RiojaLogroño, Spain; ^2^Department of Theory and Methods in Education and Psychology, School of Education and Psychology, University of NavarraPamplona, Spain; ^3^Department of Psychology, University of AlmeríaAlmería, Spain; ^4^Associated Researcher Universidad Autónoma de Chile, School of PsychologySantiago de Chile, Chile; ^5^María Inmaculada School, University of GranadaGranada, Spain; ^6^Department of Developmental and Educational Psychology, University of GranadaGranada, Spain; ^7^Department of Social Psychology Cardiff, Cardiff Metropolitan UniversityCardiff, UK

**Keywords:** resilience, self-regulation, positive youth development, at-risk youth, structural methodology

## Abstract

The ability to self-regulate behavior is one of the most important protective factors in relation with resilience and should be fostered especially in at-risk youth. Previous research has characterized these students as having behaviors indicating lack of foresight. The aim of the present study was to test the hypothetical relationship between these personal variables. It was hypothesized that self-regulation would be associated with and would be a good predictor of resilience, and that low-medium-high levels of self-regulation would lead to similar levels of resilience. The participants were 365 students -aged 15 and 21- from Navarre (Spain) who were enrolled in Initial Vocational Qualification Programs (IVQP). For the assessment, the Connor Davidson Resilience Scale (CD-RISC) and the Short Self-Regulation Questionnaire (SSRQ) were applied. We carried out linear association analyses (correlational and structural) and non-linear interdependence analyses (MANOVA) between the two constructs. Relationships between them were significant and positive. Learning from mistakes (self-regulation) was a significant predictor of coping and confidence, tenacity and adaptation, and tolerance to negative situations (resilience). Likewise, low-medium-high levels of self-regulation correlated with scores on resilience factors. Implications of these results for educational practice and for future research are discussed.

## Introduction

The European Parliament and European Council declared 2010 the European Year for Combating Poverty and Social Exclusion (Bassett and Walsh, 2011). Social exclusion is understood as the extent to which a person has or does not have a place in society (Pérez de Armiño and Eizagirre, 2005).

### Risk of social exclusion

Social exclusion is a multi-causal, dynamic, structural and multi-dimensional phenomenon (Devicienti and Poggi, 2011; Wang, 2012; Jahnukainen, 2014). This situation is often created by academic failure, which happens when students fail to finish compulsory secondary education and therefore do not meet the minimum requirements for access to the job market. In Spain and other Western countries, students who fail in the school system are one of the main groups considered to be at risk of social exclusion (Rew and Horner, 2003). This is confirmed by the indicators proposed by the Europe 2020 Strategy, which suggest that young people aged 18–24 are to be at risk of social exclusion (Eurostat, 2015). Recent research shows that the situation of this group has not improved since 2010 in Europe (Eurostat, 2013).

In recent years, Spain has launched several programs through regional and local governments, including the *Initial Vocational Qualification Programs* (IVQPs), preventive programs aimed at the educational or profesional integration of young people, for students who have not completed compulsory secondary education. These programs offer students another opportunity to study (Repper and Perkins, 2003). They provide a workframe for vocational training and they are the last chance that students have to obtain the Secondary Education qualification. The IVQP were established in 2008 in order to provide an educational opportunity for students aged 16–21 who do not have the compulsory secondary school (ESO) certificate. But these programs are also suitable for 15-year-old students when there is the possibility of dropping out of school, truancy, or rejection of the school system. The School Administration of each autonomous community within Spain is in charge of admitting students to such programs, taking into account their wishes and those of their parents.

However, it is vital that the considerable investment and effort that goes into educating young people in IVQPs should be supported by research into promoting the development of competencies. In precisely such contexts, self-regulation and resilience have been identified as key factors that can determine success or failure (Artuch, 2014). It is essential for social inclusion to be envisioned within the wider context of mental health, well-being and recovery (Repper and Perkins, 2003). In Dweck (2009): “The twenty-first century will belong to the passionate and resilient learners” (p. 9), and this probably applies to at-risk students even more than it does to those in mainstream contexts.

### Resilience and positive adaptation of students at risk

We might ask ourselves whether these students would survive better in the system if their needs, strengths or weakness were detected soon and they were prepared to overcome adversity. From the very beginnings of resilience research, emphasis has been placed on the relation between resilience and positive adaptation (Dishion and Connell, 2006). According to Nota et al. (2004), it is important to take into consideration these aspects and study their relationship with success in the education system. Analysis of resilience in high-risk groups is therefore especially important (Norris, 2014; Suria, 2016).

Over the past 50 years resilience has been studied extensively, but in the past two decades the research has broadened considerably, making incursions into social sciences, healthcare, engineering and other fields (Zolli and Healy, 2012). Since the pioneering work of Garmezy, Werner, Rutter, Luthar, Masten, Kumpfer, and others (Zolkoski and Bullock, 2012), many authors have pursued the idea of promoting resilience in children and young people who are otherwise seen as “vulnerable” (Pearce, 2011).

Empirical research establishes three critical conditions in the conceptualization of resilience: (a) growing up in, or finding oneself in an adverse situation (always subjective); (b) the availability of protective factors (internal and external), and (c) managing to adapt positively despite the experience of adversity (González-Torres and Artuch, 2014). This positive adaptation, as Fergus and Zimmerman (2005) indicate, is a resilient outcome, a way to overcome a risk. Henderson and Milstein (2003, p. 26) making clear reference to the educational sphere, define it as “…the capacity to spring back, rebound, successfully adapt in the face of adversity and develop social, academic and vocational competence despite exposure to severe stress or simply to the stress that is inherent in today's world.”

### Development of the construct

The construct *resilience* has been developed scientifically in four waves or generations of research (Zolkoski and Bullock, 2012; O'Dougherty et al., 2013; Prince-Embury and Saklosfke, 2013) and in two specific geographical settings (France and the USA). Other European authors outside France (Rutter, 2005, 2006, 2007) and Latin American authors (Kotliarenco et al., 1997) have also studied the concept, but the bulk of the literature comes from these two countries.

According to Masten's work (2004) “Resilience in developing systems: Progress and promise as the 4th wave rises,” we can point to the existence of four stages in the research about resilience. The first was interested in identifying a short list of protective/buffering factors (internal and external) when facing risk and trauma. Internal factors such as high intelligence, development of appropriate coping strategies, optimism, problem solving, self-regulation have been observed to act to protect individuals against adverse situations (Zolkoski and Bullock, 2012; García-Vesga and Domínguez-de la Ossa, 2013). On the other hand, parenting styles, family structure and cohesiveness and teacher and peer relations belong to the external protectors. Notwithstanding, it must be kept in mind that no single factor promotes resilience in isolation (Grotberg, 1995; Fergus and Zimmerman, 2005).

The second wave sought to understand how protective and risk factors interact in the process of building up resilience, and different models of resilience were developed (Fergus and Zimmerman, 2005; Becoña, 2006).

The third was interested in fostering well-being in children and young people who have grown up in adverse circumstances, placing greater effort on promoting resilience through prevention or intervention, and developing educational and healthcare policies along these lines (Catalano et al., 2004; Goldstein and Brooks, 2013; Prince-Embury and Saklosfke, 2013; Doll et al., 2014). Some of these programs, like *The Resilience Builder Program for Children and Adolescents. Enhancing Social Competence and Self-Regulation* (Karapetian et al., 2011), include self-regulation as one of the most important factors in building resilience in youth. And finally, the fourth wave is now paying increased attention to biological and genetical variables in the study of resilience, even though for decades the main focus has been on psychological or behavioral variables (Cicchetti and Curtis, 2006).

Studies about resilience have helped to change the paradigm in social and health sciences. Rather than identifying risk factors, greater attention is now devoted to identifying the individual's strengths. For this reason, resilience has been closely connected with the research objectives and goals of *Positive Psychology* (MacConville and Rae, 2012), the paradigm of *Positive Youth Development (PYD)* in the field of prevention (Lee et al., 2012), and the recommendations for promoting *Character Education* (Peterson and Seligman, 2004; Vargas and González-Torres, 2009; Goldstein and Brooks, 2013; Lerner et al., 2013; Paterson et al., 2014). These frameworks of study, which are triggering considerable scientific research and many practical applications in the clinical and educational fields, all share a belief in the plasticity of human development, and an interest in personal strengths and in seeing young people as “*resources to be developed*” (Lerner et al., 2011, p. 5). They seek to identify and understand the processes and mechanisms that underlie assets, strengths and virtues, and are interested in furthering programs and activities that not only prevent risk behaviors but also contribute to optimal development—falling within the scope of PYD (Karapetian et al., 2011).

### Assessing resilience

Despite all the advances, we still lack a clear delimitation of this construct; it continues to overlap with other related constructs such as competence or hardiness (Luthar et al., 2006; Prince-Embury, 2013). Furthermore, when dealing with a multidimensional construct, it is more difficult to reach a consensus on a clear and operational definition. The debate thus continues about what constitutes resilient behavior and how to measure it (Masten and Obradovic, 2006).

One important aspect that must be addressed in order to advance in this field is the development and trialing of measurement instruments that highlight the validity of certain psychological or external resources as resilience indicators (Fergus and Zimmerman, 2005; Windle et al., 2011; Naglieri et al., 2013). Many instruments are available today for ascertaining the extent to which a person has developed resilience at a given point in their life. Thus, we find resilience scales for children (Devereux Early Childhood Assessment: *DECA*), for young people (The Resiliency Scales for Children and Adolescents: *RSCA*; Child and Youth resilience Measure: *CYRM*; Resilience scale for Adolescents: *READ*; Resilience Scale: *RS*) and for adults (*Ego Resiliency*; Connor-Davidson Resilience Scale: *CD-RISC*) (Prince-Embury, 2013).

### Relation between resilience and self-regulation as a variable of adaptation and psychosocial protection

As we have noted, low academic achievement is among the causes that can lead a person to be at risk of social exclusion: it is a problem, because low performing students lack the basic educational qualification necessary to enter the labor market. Moreover, specifically concerning performance, Zimmerman (1990) earliest research drew our attention to the importance of self-regulation as a major factor in academic achievement. More recent research on resilience proposes the self-regulation capacity as a protective factor in this respect (Benard, 2004).

Since the pioneering work by Bandura (1991), Kanfer (1970) and others, behavioral self-regulation has become a central topic of study in general psychology and specifically in educational psychology (Zimmerman, 2008; Zimmerman and Schunk, 2011; Duckworth and Carlson, 2013; de la Fuente, 2015). Like resilience, self-regulation is an umbrella construct that incorporates many competencies (Anderman, 2011; de la Fuente et al., 2015)—self-direction, adaptability, self-management, problem-solving, critical thinking, communication and social skills—since it is a process in which the individual adopts an active role in constructing his or her destiny (requiring *will* and *skills*). Through activation, monitoring, inhibiting, preserving and adapting one's behavior, emotions, motivations, cognitive and metacognitive strategies and external resources, the person seeks to reach his/her desired objectives (Limon, 2004; Pintrich, 2004).

Self-regulation is a process that implies behavior management in three important phases (Zimmerman and Labuhn, 2012): (a) forethought and planning phase, including aspects of task analysis and setting specific task-related goals; (b) performance monitoring phase, including use of strategies and resources on the task, as well continuous examination of their effectiveness and of one's progress toward the goals established; (c) reflection on performance phase, which is evaluation of what one has done or what can be improved, managing emotions that are triggered by the results, and then using self-reflection to begin the cycle anew. This process is modulated by many variables, outstanding among which are control and self-efficacy (Torrano and González-Torres, 2004; Gardner et al., 2008; Zimmerman, 2008).

Self-regulation is a central concern in studies that examine the skills needed for students to be well prepared and successful in Twenty-first century society (Wolters, 2010; Anderman, 2011; Zimmerman and Schunk, 2011; Schleicher, 2015). de la Fuente (2015) has defined it as a behavioral meta-skill, since it makes since it makes a cross-cutting contribution in the management of several behavioral skills, especially in stressful situations.

In specialized literature in the field of resilience, self-control is considered to be a key aspect, and knowledge of strategies to improve personal self-regulation is valued (Vanistendael and Lecomte, 2006; Moilanen, 2007; Swanson et al., 2011). A large number of researchers have published work that relates self-regulation to resilience and vice versa (Nota et al., 2004; Tugade and Fredrickson, 2004; Dishion and Connell, 2006; Lerner et al., 2013). Eisenberg and Spinrad (2004) studied this relationship, indicating that self-regulation acts as a predictive factor of resilience, belonging to the individual (internal) protective factors. Similarly, Hofmann et al. (2013), Duckworth and Seligman (2005) and Mischel (2014) state that self-control (control of thoughts, emotions, impulses and behavior) encourages positive adaptation, and makes it easier to attain a happy and healthy life.

It is common for resilience scales to include self-regulation and other variables associated with it (Oshio et al., 2003; Hjemdal et al., 2006; Prince-Embury, 2008; LeBuffe et al., 2009). Studies by Buckner et al. (2003, 2009) using a single resilience instrument show that the variance in resilience explained by self-regulation may be as high as 46%. Many factors contribute to student failure and dropping out of school, but many children manage to overcome these barriers and become well-adjusted individuals (Norris, 2014).

### The aims and hypothesis

To understand any possible relation between resilience and self-regulation, we try to corroborate if self-regulation acts as a protective variable in the resilience of youths who join IVQPs. The *objectives* and *hypotheses* of this study are:

(1) To examine any relationships of association between scores in resilience and in self-regulation. A consistent correlation is assumed between the total score on both constructs and between their factors, as has been substantiated in earlier research. Additionally, we seek to identify which factor within self-regulation shows the greatest statistical significance in the association and prediction of resilience variable. As *hypotheses*, it is expected that Self-regulation factors (*goals, perseverance, decisión-making and learning from mistakes*) will have the greatest associative and predictive strength with factors from the resilience scale used in this study.

(2) To determine the interdependent relationship between levels of self-regulation and resilience. Our *hypothesis* is that a higher-medium-low level of self-regulation behavior will be accompanied by the same in the total resilience score and its components.

## Methods

### Participants

The sample consisted of 365 students from 27 schools that offer IVQPs in Navarre (Spain). Every school which imparted this type of academic training was contacted. In such programs the maximum number of students per class is 12, which means that the number of students studied in our research was reduced. Of this sample, 71.2% were male and 28.8% were female. Age distribution was as follows: 14–15 years old (19.7%), 16–17 (69.9%), 18–19 (8.5%), and 20–21 (1.9%). Regarding the schools, 61.1% of the students were enrolled in public schools, 20.5% in schools run by non-profit agencies and 18.4% in partly-subsidized private schools. The schools were spread geographically throughout Navarre (Spain). There were two IVQP modalities; 55.3% of students were enrolled in the Vocational Workshop and 44.7% were in the Basic Program. The sample is considered to be highly representative, since participation was obtained from nearly 85% of the total youth population enrolled in IVQPs during the 2011/2012 school year. The sample specific data are shown in Table 1.

**Table 1:** **Characteristics of the sample taking into account personal and contextual variables**.

**Variables**	**Category**	**N**	**%**
Gender	Men	260	71.2
	Woman	105	28.8
Age	14–15	72	19.7
	16–17	255	69.9
	18–19	31	8.5
	20–21	7	1.9
Type of center	Public	223	61.1
	Concerted	67	18.4
	Non-profit associations	75	20.5
Type of IVQP	Basic	163	44.7
	Professional workshop	202	55.3
Geographical area	City of Pamplona	204	55.9
	Mid-Zone of Navarre and Merindades of Sangüesa and Estella	59	16.2
	Ribera	89	24.4
	North of Navarre	13	3.6

### Instruments

**Figure 1:**
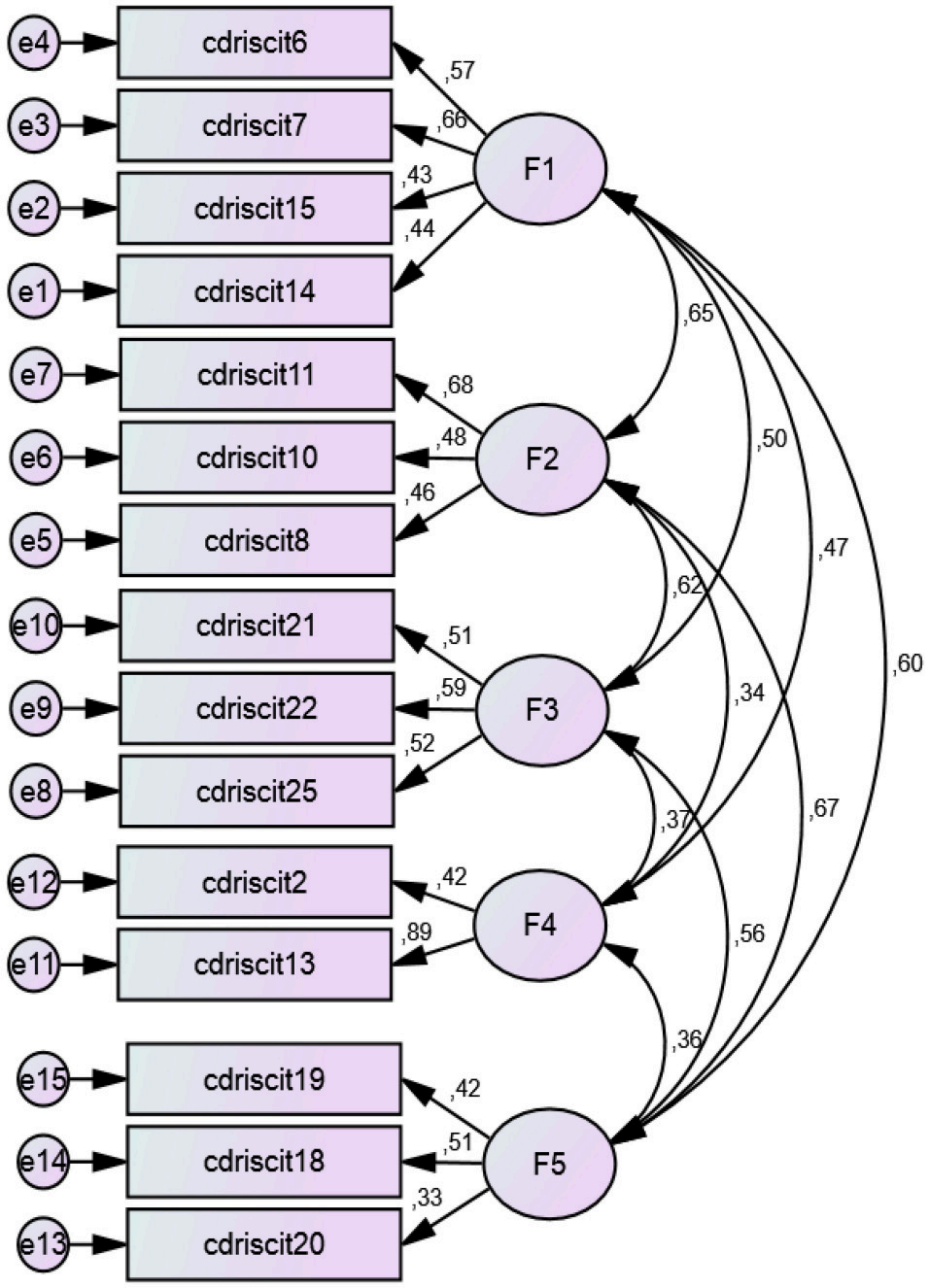
**Confirmatory factorial analysis of CD-RISC scale (*n* = 365)**. For the items, see Annex in Supplementary Meterial.

**Figure 2:**
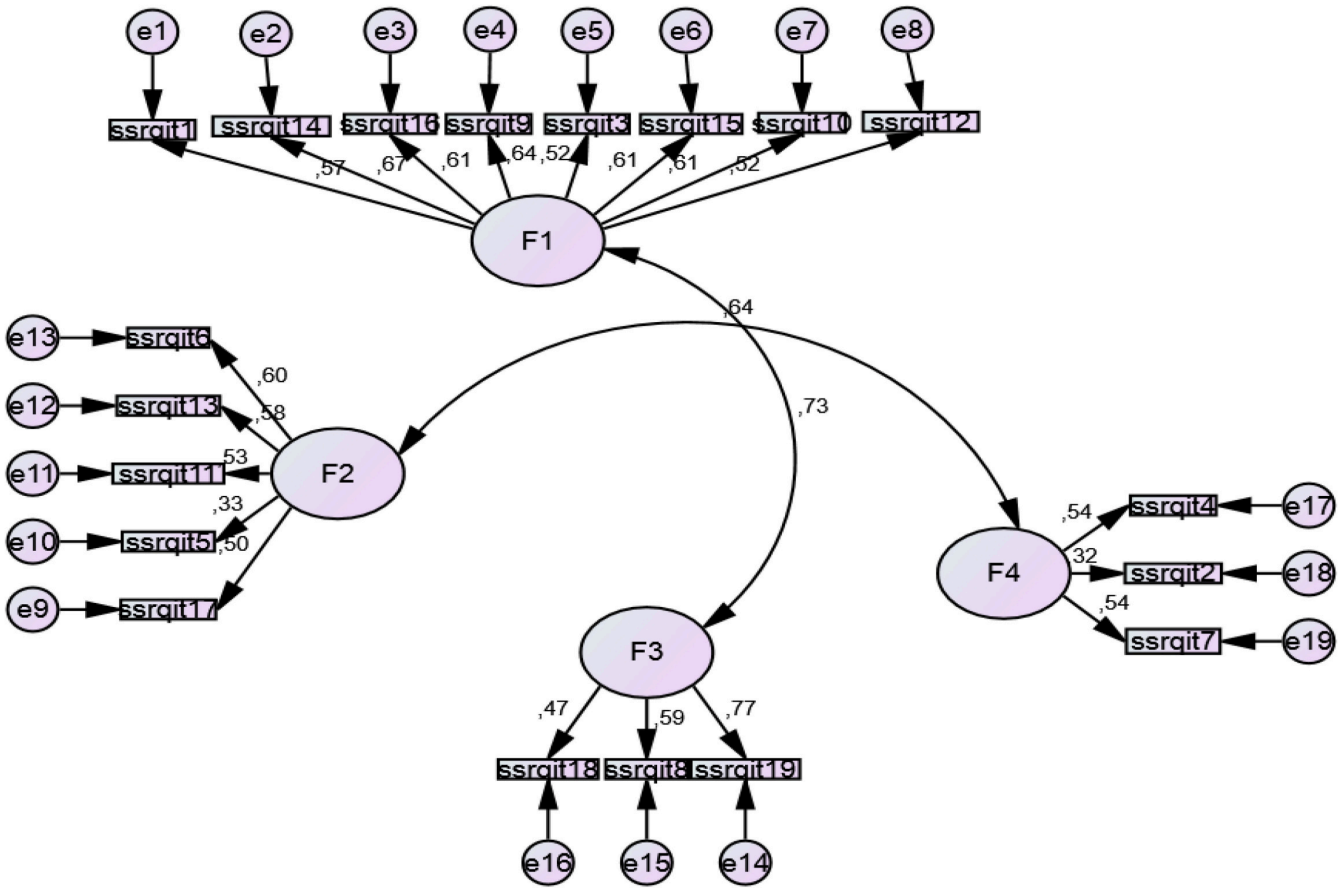
**Confirmatory structural analysis of SSRQ (*n* = 365)**. For the items, see Annex II in Supplementary Meterial.

1. The *Connor-Davidson Resilience Scale* (*CD-RISC*) in its Spanish validated version (Bobes et al., 2001-2008) was used to assess resilience. The CD-RISC scale includes a total of 25 items in its original version, grouped into 5 subscales or dimensions that measure the ability to cope with adversity. The items are scored on a scale from 0 (not true at all) to 4 (true nearly all the time). Yu and Zhang (2007) suggest that this 5-factor structure would have broad applications in psychiatric and psychological interventions and even in educational practices to nurture children with high resilience. There are few studies that have replicated the 5-factor structure (Campbell-Sills and Stein, 2007; Yu and Zhang, 2007; Yu et al., 2011; Serrano-Parra et al., 2012).
  We analyzed the internal structure of the scale. For this reason, a confirmatory factor analysis (CFA) by AMOS was conducted on the whole set of data from our sample. The default model shows a good fit (Chi-Square = 100,856, *df* = 80, *p* < 0.05; *CFI* = 0.963, *TLI* = 0.957, *IFI* = 0.968, *RFI* = 0.921 y *NFI* = 0.964; *RMSEA* = 0.027). The model proposed for this version of the scale contains 15 items with a structure of 5 factors, but different from the original version. The reformulated names of the resulting factors were: *Coping and Confidence* (Factor 1), *Tenacity and Adaptation to Change* (Factor 2), *Perception of Control, and Achievement* (Factor 3; in the original scale named “Control,” implied one's control over achieving one's own goal and getting assistance from others), *Perception of Support* (Factor 4) and *Tolerance of Negative Situations* (Factor 5). See Figure 1.
  The *reliability* coefficient of this version of the CD-RISC has Internal consistency alpha values acceptable for the total questionnaire items (α = 0.751), and an acceptable Guttman split half coefficient (α = 0.703).
2. The *Short Self-Regulation Questionnaire (SSRQ)* (Carey et al., 2004). The authors also recommend studies that analyze its factorial structure on diverse samples (Neal and Carey, 2005). This original scale has 21 items and two dimensions with Likert response choices ranging from 1 (strongly disagree) to 5 (strongly agree). Pichardo et al. (2014) validated a short version of the scale from Brown et al. (1999) in university students, finding evidence for four factors of self-regulation: *goal setting, perseverance, decision making*, and *learning from mistakes*. We also studied the internal structure of the scale in this sample through a CFA. A scale with a solution of 19 items and four factors was similar to that found in the study of Pichardo et al. (2014): factor 1 (*Goals*), factor 2 (*Perseverance*), Factor 3 (*Learning from mistakes*), and Factor 4 *(Decision making)*. This new model proposed presented a good fit (*Chi-Square* = 242,670, *df* = 150, *p* < 0.001, *CFI* = 0.929, *TLI* = 0.919, *IFI* = 0.930, RMSEA = 0.041; HOELTER = 0.290, *p* < 0.01). *Internal consistency* is acceptable for the total questionnaire items (α = 0.725) and Guttman split halves (α = 0.707). See Figure 2.

### Procedure

To initiate the research study, contact was made with all the schools and entities in Navarre that offered IVQPs in the academic year 2012-2013. Interviews were held with all the schools that agreed to participate in the research (27 of the 31 schools). A pilot study was carried out with 9 pupils to verify their understanding of each of the items on the tests and to examine the order in which they should be administered. Testing days were scheduled by telephone (first the SSRQ and then the CD-RISC, leaving a few weeks in between the two) and personal visits were made in order to apply the tests. All students were informed of the research objective and participation was voluntary. Data collection was then followed by tabulation and analysis. In all cases, there was compliance with the rules of the organizational Ethics Committees and the School Councils. The data processing was carried out according to Spanish legislation on protection of privacy.

### Data analysis

In accordance with our proposed objectives, we first carried out a *structural analysis* (association and prediction analysis). Finally, *inferential analyses* were carried out using univariate and multivariate analysis of variance (in order to determine the degree of interdependence between self-regulation, as the predicting variable and resilience, as the criterion variable). In order to classify the students into low-medium-high a prior cluster analysis was performed and the linear causal relationship between both constructs was studied, yielding mean scores for the groups at 2.81, 3.37, and 4.01, respectively. All statistical analyses were performed using statistical software from SPSS v.22 and AMOS v. 22.

## Results

### Association structural results

Statistically significant correlations were found between factors of resilience (CD-RISC) and self-regulation (SSRQ). The new model proposed presented a good fit. The default model is significant (Chi-square = 675,419, *df* = 505, *p* < 0.001; *CFI* = 0.918; *TLI* = 0.909, *IFI* = 0.920, *RFI* = 0.916 y *NFI* = 0.944; *RMSEA* = 0.030).

**Figure 3:**
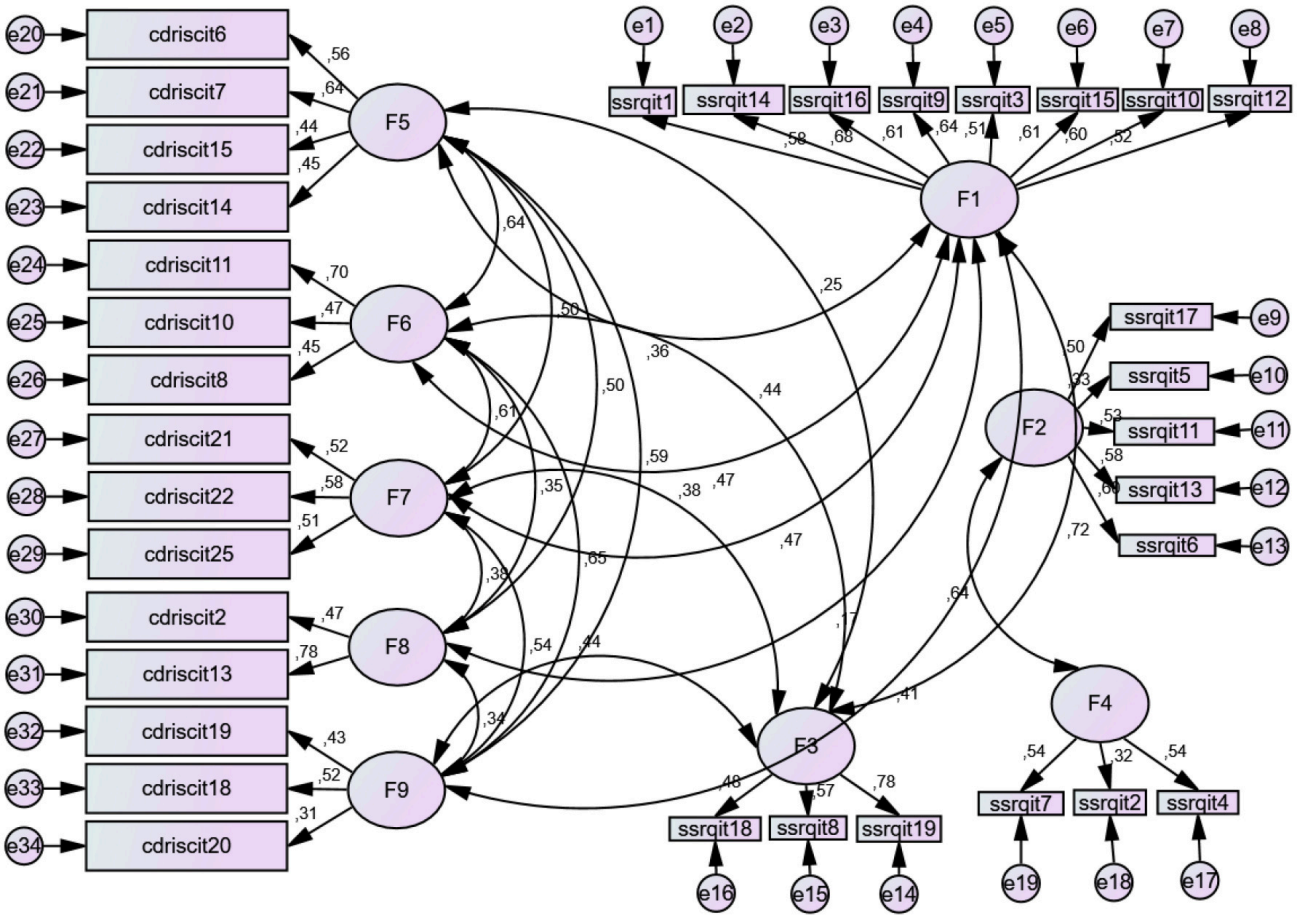
**Association relations between resilience and self-regulation factors**. Self-regulation: F1, *Goals*; F2, *Perseverance*; F3, *Learning from mistakes*; F4, *Decision making*. *Resilience*: F5, *Coping and confidence*; F6, *Tenacity and adaptation*; F7, *Perception of control and achievement*; F8, *Perception of support*; F9, *Tolerance of Negative Situations*. For the items, see Annexes I and II in Supplementary Meterial.

Self-regulation factors F1 (*goals*) and F3 (*learning from mistakes*) have the greatest associative strength with factors from resilience scale. By contrast, factors F2 (*perseverance*) and F4 (*decisión-making*) have no direct, significant relationship with any factor on this scale. See Figure 3.

### Predictive structural results

**Figure 4:**
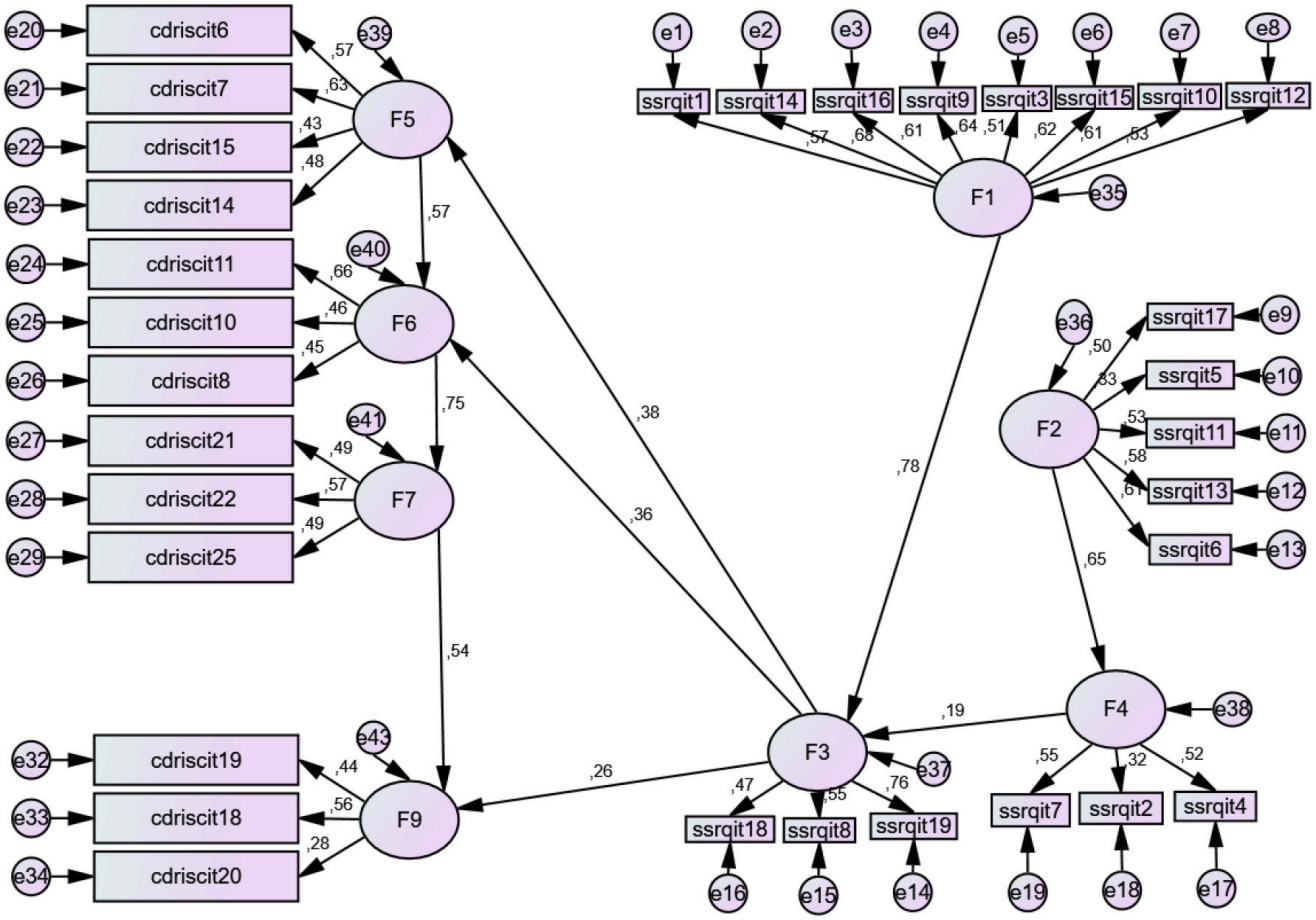
**Structural model of relations between resilience and self-regulation**. Self-regulation: F1, *Goals;* F2, *Perseverance*; F3, *Learning from mistakes*; F4, *Decision making*. Resilience: F5, *Coping and confidence*; F6, *Tenacity and adaptation*; F7, *Perception of control and achievement*; F8, *Perception of support;* F9, *Tolerance of Negative Situations*. For the items, see Annexes I and II in Supplementary Meterial.

The structural equation model used showed statistically significant relations between resilience factors (CD-RISC) and the self-regulation factors (SSRQ). The new default model is significant and presented a good fit (Chi-square = 634, 253, *df* = 454, *p* < 0.001; *CFI* = 0.910; *TLI* = 0.908, *IFI* = 0.910, *RFI* = 0.916 y *NFI* = 0.944; *RMSEA* = 0.030; *HOELTER* = 303, *p* < 0.01). As seen in the model, the self-regulation factor with the greatest predictive power over resilience is F3 (*learning from mistakes*), given that it significantly predicts factors F5 (*coping and confidence*), F6 (*tenacity*), and F9 (*tolerance of negative situations*). This factor in turn is significantly predicted by the other three self-regulation factors (F1, F2, F4). Finally, the prediction relationship between self-regulation factors F2 (*Perseverance*) and F4 (*Decision making*) and three of the resilience factors (F5, F6, and F9) is found to occur through factor F3 (*Learning from mistakes*). See Figure 4.

### Inferential non-lineal results

The ANOVA between mean total *self-regulation* (IV) and *resilience* (DV) revealed a statistically significant main effect of the first on the second variable [*F*_(2, 362)_ = 24,633, *p* < 0.001, *n*^2^ = 0.120], producing significant differences in the level of resilience according to the low-mid-high levels established for self-regulation (1 < 2 < 3, *p* < 0.001), where three homogeneous, independent subsets were obtained (Sheffé index). The MANOVA that was performed between self-regulation and the factors that make up resilience again revealed the main effect of self-regulation on the set of resilience factors [*F*_(2, 362)_ = 21.794, *p* < 0.001, *n*^2^ = 106.]. The factors where self-regulation produced the greatest effect were *Coping and Confidence* [*F*_(2, 362)_ = 21,794, *p* < 0.001, *n*^2^ = 0.107] and *Tenacity and Adaptation to Change* [*F*_(2, 362)_ = 12,803, *p* < 0.001, *n*^2^ = 0.066], with statistical significant differences among the levels 1 < 2 < 3, *p* < 0.01, in both cases. The least effect was seen on the *tolerance of negative situations* factor [*F*_(2, 362)_ = 4,722, *p* < 0.05, *n*^2^ = 0.025], with differences only between the extreme groups (3 > 1, *p* < 0.05). There are no differences in the *perception of support* factor. See Table 2.

**Table 2:** **Mean values for resilience, according to level of prior self-regulation (*n* = 365)**.

	**Self-regulation levels**		
	**Low *n* = 89**	**Mid *n* = 187**	**High *n* = 89**
F1. Coping and confidence	3.44 (0.76)	3.65 (0.66)	4.09 (0.64)
F2. Tenacity and adaptation to change	3.57 (0.68)	3.73 (0.71)	4.08 (0.64)
F3. Perception of control and achievement	3.56 (0.85)	3.84 (0.76)	4.10 (0.71)
F4. Perception of support	3.70 (0.99)	3.78 (0.96)	4.00 (0.87)
F5. Tolerance of negative situations	3.24 (0.72)	3.37 (0.69)	3.57 (0.76)
Total resilience	3.48 (0.51)	3.67 (0.48)	3.98 (0.41)

## Discussion

In the studies that have been carried out over the years about social exclusion, one of the main risk factors is dropping out of education. Students who fail at school and do not get a basic qualification might suffer social exclusion due to their difficulties in gaining access to the labor market. This research is expected to contribute to our knowledge of certain aspects of social exclusion, and to help prevent dropouts. One of the variables chosen was self-regulation since, according to the research, it has been associated with a good (or high) performance (Nota et al., 2004). Over the past decades, several research studies have pointed out the importance of studying protective factors and attributes that define the resilient personality. Self-regulation occupies a central place among these (Eisenberg and Spinrad, 2004; Novoa, 2014).

Also, according to Nota et al. (2004) there is an important relationship between self-regulation, resilience and academic achievement. The present investigation is a relevant contribution to this line of current research—within the realm of preventive, educational psychology—focusing on analysis of psychological constructs that are important for bearing adversity in everyday life, and for mental health education (Henderson and Milstein, 2003).

### Levels and relationships between resilience and self-regulation

The first objective and hypothesis, referring to *associations* between the two constructs *and predictive structural analysis*, was partly achieved: there were positive, statistically significant correlations between some of the factors on the two scales. Most notably, the factors *goals* and *learning from mistakes* were significantly related to resilience factors.

Among the different factors that influence resilience, self-regulation was confirmed as important, but there are other personal variables that also play their part (Gardner et al., 2008; Buckner et al., 2009). Self-regulation models indicate the importance of the planning and goal-setting phase in the process of self-regulating one's behavior (Zimmerman, 2008). However, our research shows that, although the *goals* factor is essential for developing resilience, its predictive value is indirect and takes place through the *learning from mistakes* factor, as mentioned above. Thus, for this sample of young people, this is the factor that best predicts the three factors of resilience: *coping and confidence, tenacity, and adaptation to change*, and *tolerance of negative situations*. This result has interesting practical implications indicating that, in order to improve resilience through self-regulation in students in socially at-risk situations, it is very important to work on *goal-setting* and above all, *learning from mistakes*. Both aspects are essential to learning, as recently noted in the document “Top 20 principles from psychology for pre K–12 teaching and learning” published by American Psychological Association (2015). Directing and modifying behavior when mistakes are detected is the essential element of metacognition that is at the center of self-regulated behavior. Our data indicate persevering in the search for solutions predicts tenacity (resilience). Along these lines, Dweck et al. (2011) in their paper *Academic tenacity*, indicate that in order to enhance it, it is important to instill a growth mindset about intelligence where failures and mistakes are seen as natural part of the learning process.

The second objective and hypothesis, concerning interdependent non-linear relations between levels on the two scales, has been validated consistently. The results conclusively establish that low-mid-high levels of self-regulation are accompanied by comparable differences in three levels of resilience and independent groups are established with statistically significant differences between them. This evidence demonstrates a consistent relationship between the two constructs, as corroborated by other studies (Eisenberg and Spinrad, 2004; Dishion and Connell, 2006).

### Limitations

Certain *limitations* must be kept in mind. For one, though our sample is broad and representative of the population of young people who are enrolled in IVQP, it is not uniform in age, gender and cultural background.

### Conclusions, implications, and prospects for research

Findings from this research show progress in our understanding of resilient and self-regulatory behavior in adolescent students at risk, and provides empirical support for the theoretical relations of association and interdependence between the two constructs. Moreover, we offer data on concurrent validation of two standardized assessment instruments that address these constructs.

Additionally, these results offer empirical support for the theory regarding relations between resilience and self-regulation (Lee et al., 2012). Moreover, although these results provide data on the convergent validity of the two instruments used, certain factors on the scales that did not present the expected relationships must be examined more closely. The present investigation makes contributions that will help us to advance in the development of valid and reliable measures that are necessary to progress in the theoretical and applied aspects of this field (Windle et al., 2011).

As to *implications* for education, the findings from this research can help us to better understand how to nurture a *resilient mindset* (Goldstein and Brooks, 2013) in young people enrolled in IVQPs, to achieve better adaptation and avoid social exclusion. These results may also serve as a guide about how to help these students both inside and outside the classroom, such as creating supportive “ecological niches” (Henderson and Milstein, 2003) that encourage their sense of self-efficacy and their problem-solving ability.

In this sense it would be beneficial to work on certain essential aspects of behavior self-regulation. Particularly, setting realistic goals and learning from mistake. It is the case in proven effectiveness prevention and intervention programs, such as the PENN Resilience Program (PRP) (Reivich et al., 2013), or the Resilience Builder Program for Children and Adolescents. Enhancing Social Competence and Self-Regulation (Karapetian et al., 2011), the *PATHS* Project (Promoting Alternative Thinking Strategies), I Can Problem Solve (ICPS) (Shure, 2001) or in Spain, the Scholastic and social well-being program: a promotion program for adolescents (López-Sánchez et al., 2007). On this point -and as we underlined in the second and third stage of the research-, self-regulation has been an important variable which is considered as a protective factor or element in a risk situation, and has been included in resilience programmes. We emphasize that by working on self-regulation skills with students at risk, we will encourage their resilient capacity. Then, they will not give up when they have to deal with difficulties (Masten and Obradovic, 2006) and they will be able to improve their academic achievement necessary to get access to the job market, and will not suffer social exclusion. Consequently, we consider the research presented here to be valuable in helping us to understand the characteristics of students at risk that enroll in the IVQPs. These results show the importance of working on student strengths that go beyond the academic or technical areas and which can help them cope positively with the adverse situations in which they live, so that they can build an optimistic life plan, based on competencies of resilience such as self-regulation.

## Author contributions

RA: substantially contributed to the conception and the design of the work. MG: manuscript preparation; ML and MF contributed to the acquisition of data JD: study design, statistical analysis; RA prepared the draft and MG and MV reviewed it critically and gave important intellectual content RA, MG and JD: worked for the final approval of the version to be published.

## Funding

Motivational-affective strategies of personal self-regulation and coping with stress in the university teaching-learning process. Ref. EDU2011-24805 (2012-2015). Ministry of Economy and Competitiveness (Spain) and FEDER Found (EU).

### Conflict of interest statement

The authors declare that the research was conducted in the absence of any commercial or financial relationships that could be construed as a potential conflict of interest.
