# Supplementary material for: Relationship between Resilience and Self-regulation: A Study of Spanish Youth at Risk of Social Exclusion
Source: Front Psychol. 2017 Apr 20;8:612. doi: 10.3389/fpsyg.2017.00612 (PMC5397523; doi:10.3389/fpsyg.2017.00612)
Supplement: Supplementary file 1 [file Table1.doc]

*Anex 1*. CD-RISC ABREVIATED

| Resilience Scale CD-RISC |
| --- |

|  | Ítem and factor | M (DT) |
| --- | --- | --- |
| F1 Coping and Confidence | 6. I try to see the humorous side of things when I am faced with problems. | 3.66 (1.1) |
|  | 7. Having to cope with stress can make me stronger. | 3.94 (.99) |
|  | 14. Under pressure, I stay focused and think clearly. | 3.3 (1.07) |
|  | 15. I prefer to take the lead in solving problems, rather than letting others make all the decisions. | 3.92(1.09) |
| F2 Tenacity and Adaptation to change | 8. I tend to bounce back after illness, injury, or other hardships. | 3.86(1.02) |
|  | 10. I give my best effort, no matter what the outcome may be. | 3.55(1.02) |
|  | 11. I believe I can achieve my goals, even if there are obstacles. | 3.93(.93) |
| F3 Perception of Control and Achievement | 21. I have a strong sense of purpose in life. | 3.94(1.09) |
|  | 22. I feel in control of my life. | 3.63(1.06) |
|  | 25. I take pride in my achievements. | 3.93(1.13) |
| F4 Perception of Support | 2. I have at least one close and secure relationship which helps me when I am stressed. | 3.70(1.19) |
|  | 13. During times of stress/crisis, I know where to turn for help. | 3.92(1.11) |
| F5 Tolerance to negative situations | 18. I can make unpopular or difficult decisions that affect other people, if it is necessary. | 3.28(1.03) |
|  | 19. I am able to handle unpleasant or painful feelings like sadness, fear, and anger. | 3.53(1.15) |
|  | 20. In dealing with life's problems, sometimes you have to act on a hunch, without knowing why. | 3.36(1.07) |
