# Supplementary material for: Relationship between Resilience and Self-regulation: A Study of Spanish Youth at Risk of Social Exclusion
Source: Front Psychol. 2017 Apr 20;8:612. doi: 10.3389/fpsyg.2017.00612 (PMC5397523; doi:10.3389/fpsyg.2017.00612)
Supplement: Supplementary file 2 [file Table2.doc]

*Anex 2*. SRQ. ABREVIATED

| Short Self regulation scale SSRQ | | |
| --- | --- | --- |
|  | Item and factor | M (DT) |
| F1 Goals | 1. I usually keep track of my progress toward my goals | 3.67(.98) |
|  | 3. I am able to accomplish goals I set for myself | 3.84(.93) |
|  | 9. I have personal standards, and try to live up to them | 3.75(.93) |
|  | 10. As soon as I see a problem or challenge, I start looking for possible solutions | 3.81(1) |
|  | 12. When I'm trying to change something, I pay a lot of attention to how I'm doing | 3.73(.99) |
|  | 14. I set goals for myself and keep track of my progress | 3.54(.97) |
|  | 16. If I make a resolution to change something, I pay a lot of attention to how I’m doing | 3.68(.99) |
|  | 15. I can usually find several different possibilities when I want to change something | 3.63(.94) |
| F2 Perseverance | 5. It's hard for me to notice when I've “had enough” (alcohol, food, sweets) | 3.15(1.47) |
|  | 6. I have trouble following through with things once I've made up my mind to do something. | 3.05(1.19) |
|  | 11. Have a hard time setting goals for myself | 3.10(1.14) |
|  | 13. I have trouble making plans to help me reach my goals | 3.06(1.06) |
|  | 17. Often I don't notice what I'm doing until someone calls it to my attention | 2.70(1.21) |
| F3 Learning from mistakes | 8. I usually only have to make a mistake one time in order to learn from it | 3.83(1.16) |
|  | 18. I usually think before I act. | 3.50(1.21) |
|  | 19. I learn from my mistakes | 3.81(1.05) |
| F4 Decision making | 2. I don't notice the effects of my actions until it's too late | 2.84(1.09) |
|  | 4. I put off making decisions | 2.97(1.13) |
|  | 7. I don’t seem to learn from my mistakes | 3.32(1.25) |
